# Osteoporosis Health Beliefs of Women with Increased Risk of the Female Athlete Triad

**DOI:** 10.1155/2014/676304

**Published:** 2014-03-09

**Authors:** Vu H. Nguyen, Ze Wang, Stephanie M. Okamura

**Affiliations:** ^1^Public Health Program, Department of Health Sciences, University of Missouri, Columbia, MO 65211, USA; ^2^Department of Educational, School, and Counseling Psychology, University of Missouri, Columbia, MO 65211, USA; ^3^Public Health Program, University of Missouri, Columbia, MO 65211, USA

## Abstract

Women with increased risk of the female athlete triad (Triad) are more susceptible to osteoporosis compared to other women. The study included 65 women with increased risk of the Triad who had their osteoporosis health beliefs measured to assess their concern for the disease. Participants were female collegiate cross-country runners at different levels of competition, including National Association of Intercollegiate Athletics (NAIA) and National Collegiate Athletic Association (NCAA) Divisions III, II, and I. Although these participants have an increased risk of the Triad and are more susceptible to osteoporosis, on a scale of 1 to 5, results showed that they had low to moderate perceived susceptibility to osteoporosis with a mean score as high as 2.81 and moderate perceived severity of osteoporosis with a mean score as high as 3.38. A statistically significant difference in perceived susceptibility to osteoporosis was found between female collegiate cross-country runners in the NAIA and those in the NCAA DIII. Reasons that could explain relatively low levels of concern for osteoporosis in female collegiate cross-country runners and reasons for significant differences in perceived susceptibility to osteoporosis are given, and recommendations for health education and intervention to help care for this population are provided.

## 1. Introduction

Osteoporosis is a severe disease that reduces both the quality [1] and quantity [2] of life. The disease is characterized as having decreased bone strength leading to increased fracture risk, and it is clinically diagnosed as having bone mineral density (BMD) 2.5 standard deviations below the adult peak mean [3]. The disease increases morbidity and mortality, and it affects hundreds of millions of individuals worldwide [4]. While osteoporosis can happen to anyone of either gender, women have higher rates of prevalence for the disease, especially women of Caucasian and Asian ethnicity, and some women may be at even higher risk of osteoporosis due to the female athlete triad.

The female athlete triad (Triad) is a syndrome that is an interrelationship between (1) low energy availability, (2) irregular menstrual cycles, and (3) osteoporosis [5]. Low energy availability can result from eating disorders, such as anorexia nervosa and bulimia nervosa, in which calorie consumption is limited or purged, respectively. Irregular menstrual cycles can result in amenorrhea, which is an absence of menstrual cycles for at least three months. Women having the conditions of eating disorders and/or amenorrhea can have reduced BMD that decreases bone health and strength, which increases their risk of osteoporosis.

Women who are at higher risk of the Triad are athletes who engage in physical activity for prolonged periods while restricting their diets to maintain or lose body weight. High school female athletes have low prevalence of the Triad at just over 1% [6], but college female athletes have higher prevalence of the Triad, as nearly two-thirds of college female runners are affected with at least one component of the Triad [7]. In particular, female collegiate cross-country runners have increased prevalence of the Triad due to their high volume of physical activity and necessity to maintain or lower body weight. In a study of female collegiate cross-country runners, nearly one-fifth had previous or current eating disorders, nearly a quarter had irregular menstrual cycles, and nearly a third had inadequate calcium intake [8]. Compared to their male counterparts, female collegiate athletes are at higher risk of eating disorders, such as anorexia nervosa and bulimia nervosa [9], and female collegiate cross-country runners have also been found to lack nutritional knowledge critical to preventing related health issues [10]. In addition, female collegiate cross-country runners' training has been shown to actually decrease their BMD, and they are at greater risk of lower bone mass than other athletes [11], which increases their risk of osteoporosis.

Female collegiate cross-country runners have an increased risk of the Triad, and while that leads to an increased risk of osteoporosis, their concern for the disease is unknown. To investigate their possible concern for osteoporosis, osteoporosis health beliefs based on Rosenstock's [12] health belief model (HBM), such as the perceived susceptibility to osteoporosis and the perceived severity of osteoporosis, were measured and analyzed.

## 2. Materials and Methods

### 2.1. Participants and Procedures

To investigate female collegiate cross-country runners' osteoporosis health beliefs, the research university's Institutional Review Board granted approval for the study. Participants were a convenience sample of runners from women's cross-country teams from various colleges and universities in the same state as the research university, which was located in the midwest region of the United States (US). There were a total of 65 female collegiate cross-country runners who participated in this study, and they competed at different levels (see Table 1). The majority of the participants were of Caucasian ethnicity and between 19.5 and 20.7 years old. Fifteen participants competed in the National Association of Intercollegiate Athletics (NAIA), which is a lower level of organized competition and is comprised of smaller colleges and universities in the US. Fifty participants competed in the National Collegiate Athletic Association (NCAA), which is a higher level of competition than the NAIA. These participants included 26 participants competing in its lowest level, Division III (DIII); 10 participants competing in Division II (DII); 14 participants competing in the NCAA's highest level and the highest level of competition in the US, Division I (DI). Each participant completed a survey consisting of a written letter of consent, demographic information, and a scale to measure osteoporosis health beliefs. The surveys were returned to the researcher.

### 2.2. Measures

Osteoporosis health beliefs were measured with the first 12 items of the Osteoporosis Health Belief Scale (OHBS). The OHBS is an instrument developed by Kim et al. [13] to measure each of the constructs of the HBM. The OHBS is divided into two subscales, the OHB-Exercise and OHB-Calcium subscales. In the OHB-Exercise, for reliability, Cronbach's alpha coefficient for perceived susceptibility to osteoporosis is 0.80 and for perceived severity of osteoporosis is 0.65. For validity, with construct validity and factor analysis for each item, perceived susceptibility to osteoporosis accounted for 15.9% and perceived severity of osteoporosis accounted for 12.1% of total variance. In the OHB-Calcium, for reliability, Cronbach's alpha coefficient for perceived susceptibility to osteoporosis is 0.80 and perceived severity of osteoporosis is 0.65. For validity, with construct validity and factor analysis for each item, perceived susceptibility to osteoporosis accounted for 14.4% and perceived severity of osteoporosis accounted for 12.4% of total variance.

The first 12 items of the OHBS were used in this study to measure (1) perceived susceptibility to osteoporosis (items 1–6) and (2) perceived severity of osteoporosis (items 7–12). Items for perceived susceptibility to osteoporosis included the following. “(1) Your chances of getting osteoporosis are high.” “(2) Because of your body build, you are more likely to develop osteoporosis.” “(3) It is extremely likely you will get osteoporosis.” “(4) There is a good chance that you will get osteoporosis.” “(5) You are more likely than the average person to get osteoporosis.” “(6) Your family history makes it more likely that you will get osteoporosis.” Items for perceived severity of osteoporosis included the following. “(7) The thought of having osteoporosis scares you.” “(8) If you had osteoporosis, you would be crippled.” “(9) Your feelings about yourself would change if you got osteoporosis.” “(10) It would be very costly if you got osteoporosis.” “(11) When you think about osteoporosis you get depressed.” “(12) It would be very serious if you got osteoporosis.” The responses to each of the 12 items are a 5-point Likert scale: SD (Strongly Disagree) = 1, D (Disagree) = 2, N (Neutral) = 3, A (Agree) = 4, and SA (Strongly Agree) = 5. Scores for (1) perceived susceptibility to osteoporosis, and (2) perceived severity of osteoporosis, were calculated by the average score of the 6 items for each construct and ranged from 1 to 5.

### 2.3. Statistical Analysis

Descriptive statistics for each construct measured, (1) perceived susceptibility to osteoporosis, and (2) perceived severity of osteoporosis, in addition to each of the 12 items of the OHBS, were reported for all participants and each group. One-way analysis of variance was conducted to find statistically significant differences between groups.

## 3. Results

Table 2 shows the scores for perceived susceptibility to osteoporosis with each item and perceived severity of osteoporosis with each item for all participants and for each level of competition. Perceived susceptibility to osteoporosis was low to moderate for all levels of competition with a mean as low as 2.06 for NAIA and as high as 2.80 for NCAA DIII. Perceived severity of osteoporosis was moderate for all levels of competition with mean scores ranging as low as 3.02 for NAIA and as high as 3.38 for NCAA DIII.

The reliability, calculated as Cronbach's alpha, of the six items measuring perceived susceptibility to osteoporosis was 0.90 from this sample. The reliability of the six items measuring perceived severity of osteoporosis was 0.78 from this sample. There was a positive, low, and statistically significant correlation between the two measures, *r* = 0.319 and *P* = 0.01.

Table 3 shows mean scores by competition level. Perceived susceptibility to osteoporosis scores was between “disagree” to “neutral,” meaning that this sample generally perceived that they had low to moderate susceptibility to osteoporosis. Perceived severity of osteoporosis scores was between “neutral” and “agree,” higher than for perceived susceptibility to osteoporosis. This sample perceived that osteoporosis would be moderately severe if they would have osteoporosis. There was a significant competition level effect on perceived susceptibility, *F*(3,61) = 3.33, *P* < 0.03. Competition level accounted for 14% of the variance in perceived susceptibility (*η*_partial_^2^ = 0.14). Based on pairwise comparisons, a significant difference existed between those who competed at the NAIA level and those who competed at the NCAA DIII level. Those competing at the NAIA level had significantly lower perceived susceptibility to osteoporosis than those who competed at the NCAA DIII level. There was no significant competition level effect on perceived severity of osteoporosis, as participants competing at different levels did not differ significantly on perceived severity of osteoporosis.

## 4. Discussion

To explain why female collegiate cross-country runners have low to moderate perceived susceptibility to osteoporosis and moderate perceived severity of osteoporosis, Nguyen et al. [14] found that it is likely due to the distal time of onset for osteoporosis that college-aged women have minimal concerns for the disease. Female collegiate cross-country runners may be more concerned with the proximal results of improved athletic performance that the distal consequences of osteoporosis are of minimal concern.

For both perceived susceptibility to osteoporosis and perceived severity of osteoporosis, participants in NAIA had the lowest scores, while participants in NCAA DIII had the highest scores. Whereas the difference between the two groups was not statistically significant at the .05 level in perceived severity of osteoporosis, there was a significant difference between them in perceived susceptibility to osteoporosis. It is not clear why female collegiate cross-country runners who competed in the NCAA DIII perceived themselves more susceptible to osteoporosis than those who competed in NAIA. It might be that the NAIA is the lowest level of collegiate competition, and for female collegiate cross-country runners who compete in the NAIA, perhaps the lack of higher-level competition produces less intense training and need for disordered eating to enhance performance. This may have caused lower levels of perceived health consequences such as a lowered perceived risk of osteoporosis. The NCAA is a higher level of collegiate competition than the NAIA, but female collegiate cross-country runners in NCAA DIII are competing in its lowest level. For them, there may be a need to enhance performance with increased training and/or disordered eating to compete at a higher level within the NCAA. Athletic scholarships are not given in NCAA DIII, but they are given in NCAA DII and DI. Perhaps athletes competing in NCAA DIII desire to move up to NCAA DII or DI to earn an athletic scholarship, even if engaging in behaviors that enhanced performance would compromise health.

We found that osteoporosis concerns for female collegiate cross-country runners in all four groups were relatively low. There is a need to increase concern for osteoporosis for all female collegiate cross-country runners, as they are at an increased risk of the Triad and of osteoporosis.

To prevent and treat those at risk or affected with the Triad, the American College of Sports Medicine (ACSM) in a position stand has provided guidelines for prevention and treatment. Prevention tactics should focus on health education and counseling, regular screenings, and medical support for athletes to increase health knowledge that could prevent the development of this syndrome. Athletes of all ages should be screened for Triad symptoms during regular health examinations. Health practitioners should document related conditions, such as low bone mineral density levels, and conduct laboratory tests, if necessary, to determine the cause. Chronic injuries, such as stress fractures, may also require further examination. Nutrition counseling and mental health care should be offered to those displaying disordered eating habits, whether or not they meet strict clinical criteria for an eating disorder, with treatment focused on increasing caloric intake to replenish energy and resume regular menstrual activity [5].

The ACSM also recommends that athletes must agree to comply with treatment and prioritize health and safety, even if athletic performance diminishes, in order to continue competing. This may involve modifying training plans and limiting competitions. Given the necessity of compliance, athletes who resist treatment should be considered for removal from the sport. In addition, multidisciplinary support from family, coaches, and particularly from professionals such as physicians, registered dietitians, and mental health specialists may be needed in order for athletes to recover from the syndrome. Furthermore, the ACSM also urges sports organizations to discourage restrictive eating through sports-specific policies and penalties in order to prevent and treat the Triad [5].

This study was not without limitations. As with all self-reported data, when completing the surveys, social desirability and random responding by the participants were a concern. Female collegiate cross-country runners from various colleges and universities in one state participated in this study, and results may not reflect osteoporosis health beliefs of female collegiate cross-country runners from other states or regions in the country. Another limitation is the relatively small sample size, which might have limited the statistical power to detect more significant differences.

## 5. Conclusion

This study demonstrated that women who may be at higher risk of the Triad and of osteoporosis, such as female collegiate cross-country runners, did not perceive themselves as susceptible to osteoporosis nor believe it is a severe disease if they were to be diagnosed with it. More research is needed, but the results of this study show that these women should be provided adequate health education as they may be at increased risk of osteoporosis, and interventions should be considered to help reduce their chances of acquiring the disease.

## Figures and Tables

**Table 1:** Demographic information.

Levels of competition	Number of participants	Ethnicity	Age mean
NAIA	15	93.3% Caucasian	20.13 (1.46)
NCAA Division III	26	88.5% Caucasian	19.58 (1.21)
NCAA Division II	10	100% Caucasian	20.70 (1.16)
NCAA Division I	14	78.6% Caucasian	19.50 (1.09)

Standard deviations for age are in parentheses.

**Table 2:** Osteoporosis health belief average and individual item scores for levels of competition.

Osteoporosis health belief and individual items	NAIA	NCAA Division III	NCAA Division II	NCAA Division I
Perceived susceptibility to osteoporosis (average of items 1–6)	**2.06 (0.51)**	**2.81 (0.94)**	**2.65 (1.03)**	**2.24 (0.66)**
(1) Your chances of getting osteoporosis are high.	2.13 (0.74)	2.92 (1.04)	2.80 (0.92)	2.43 (1.09)
(2) Because of your body build, you are more likely to develop osteoporosis.	2.33 (0.90)	2.68 (1.22)	3.00 (1.25)	2.43 (1.02)
(3) It is extremely likely you will get osteoporosis.	2.00 (0.54)	2.16 (0.90)	2.20 (1.14)	2.21 (0.58)
(4) There is a good chance that you will get osteoporosis.	2.07 (0.46)	2.72 (0.89)	2.60 (1.17)	2.29 (0.73)
(5) You are more likely than the average person to get osteoporosis.	1.93 (0.80)	3.32 (1.03)	2.60 (1.17)	2.36 (1.08)
(6) Your family history makes it more likely that you will get osteoporosis.	1.87 (0.83)	2.56 (1.00)	2.70 (1.42)	1.71 (0.47)
Perceived severity of osteoporosis (average of items 7–12)	**3.02 (0.83)**	**3.38 (0.61)**	**3.07 (0.30)**	**3.27 (0.67)**
(7) The thought of having osteoporosis scares you.	3.60 (1.35)	4.12 (0.95)	3.70 (0.48)	3.64 (1.01)
(8) If you had osteoporosis, you would be crippled.	2.33 (0.98)	2.58 (0.76)	2.50 (0.85)	2.79 (0.98)
(9) Your feelings about yourself would change if you got osteoporosis.	2.60 (1.06)	3.12 (0.95)	2.60 (1.07)	3.21 (1.37)
(10) It would be very costly if you got osteoporosis.	3.53 (0.64)	3.77 (0.82)	3.60 (0.70)	3.57 (0.76)
(11) When you think about osteoporosis you get depressed.	2.47 (1.19)	2.81 (1.17)	2.50 (0.85)	2.64 (0.93)
(12) It would be very serious if you got osteoporosis.	3.60 (1.06)	3.88 (0.82)	3.50 (0.53)	3.79 (0.58)

Scores range from 1 to 5. Standard deviations are in parentheses.

**Table 3:** Perceived susceptibility to and perceived severity of osteoporosis by level of competition and analysis of variance results.

Level of competition	Perceived susceptibility to osteoporosis	Perceived severity of osteoporosis
	Mean (SD)	Mean (SD)
NAIA (*n* = 15)	2.06 (0.51)^a^	3.02 (0.83)
NCAA Division III (*n* = 26)	2.81 (0.94)^a^	3.38 (0.61)
NCAA Division II (*n* = 10)	2.65 (1.03)	3.07 (0.30)
NCAA Division I (*n* = 14)	2.24 (0.66)	3.27 (0.67)
*F*(3,61)	3.33	1.21
*P*	0.03	0.32
Effect size (*η*_partial_^2^)	0.14	0.06

Note: Values in the same column with the same superscript are statistically different at 0.05 level after Bonferroni adjustment.
